# Iron Imaging as a Diagnostic Tool for Parkinson's Disease: A Systematic Review and Meta-Analysis

**DOI:** 10.3389/fneur.2020.00366

**Published:** 2020-05-28

**Authors:** Nadya Pyatigorskaya, Clara B. Sanz-Morère, Rahul Gaurav, Emma Biondetti, Romain Valabregue, Mathieu Santin, Lydia Yahia-Cherif, Stéphane Lehéricy

**Affiliations:** ^1^Institut du Cerveau et de la Moelle épinière (ICM), Centre de NeuroImagerie de Recherche (CENIR), ICM, Paris, France; ^2^Sorbonne Université, UPMC Univ Paris 06, UMR S 1127, CNRS UMR 7225, ICM, Paris, France; ^3^Assistance Publique Hôpitaux de Paris, Service de neuroradiologie, Hôpital Pitié-Salpêtrière, Paris, France

**Keywords:** substantia nigra, iron, Parkinson's disease, QSM, SWI, R2^*^

## Abstract

**Background:** Parkinson's disease (PD) is a progressive neurodegenerative disease whose main neuropathological feature is the loss of dopaminergic neurons of the substantia nigra (SN). There is also an increase in iron content in the SN in postmortem and imaging studies using iron-sensitive MRI techniques. However, MRI results are variable across studies.

**Objectives:** We performed a systematic meta-analysis of SN iron imaging studies in PD to better understand the role of iron-sensitive MRI quantification to distinguish patients from healthy controls. We also studied the factors that may influence iron quantification and analyzed the correlations between demographic and clinical data and iron load.

**Methods:** We searched PubMed and ScienceDirect databases (from January 1994 to December 2019) for studies that analyzed iron load in the SN of PD patients using T2^*^, R2^*^, susceptibility weighting imaging (SWI), or quantitative susceptibility mapping (QSM) and compared the values with healthy controls. Details for each study regarding participants, imaging methods, and results were extracted. The effect size and confidence interval (CI) of 95% were calculated for each study as well as the pooled weighted effect size for each marker over studies. Hence, the correlations between technical and clinical metrics with iron load were analyzed.

**Results:** Forty-six articles fulfilled the inclusion criteria including 27 for T2^*^/R2^*^ measures, 10 for SWI, and 17 for QSM (3,135 patients and 1,675 controls). Eight of the articles analyzed both R2^*^ and QSM. A notable effect size was found in the SN in PD for R2^*^ increase (effect size: 0.84, 95% CI: 0.60 to 1.08), for SWI measurements (1.14, 95% CI: 0.54 to 1.73), and for QSM increase (1.13, 95% CI: 0.86 to 1.39). Correlations between imaging measures and Unified Parkinson's Disease Rating Scale (UPDRS) scores were mostly observed for QSM.

**Conclusions:** The consistent increase in MRI measures of iron content in PD across the literature using R2^*^, SWI, or QSM techniques confirmed that these measurements provided reliable markers of iron content in PD. Several of these measurements correlated with the severity of motor symptoms. Lastly, QSM appeared more robust and reproducible than R2^*^ and more suited to multicenter studies.

## Introduction

Parkinson's disease (PD) is a progressive neurodegenerative disease whose main neuropathological characteristic is the loss of dopaminergic neurons of the substantia nigra (SN) pars compacta (SNc) (1). Degeneration of dopaminergic neurons in the SN of PD patients is accompanied by an increase in iron content. Iron is necessary for body homeostasis, oxygen transport, or central nervous system development, but its capacity of producing reactive oxygen species that lead to stress oxidation can have a deleterious effect on the SN of PD patients. Iron also plays an important role in the neurodegenerative processes associated with PD (2–4).

Iron is a paramagnetic element that induces magnetic field inhomogeneities, that is, differences in the local magnetic field relative to the mainly diamagnetic surrounding brain tissues. Iron-induced local field inhomogeneities increase spin–spin interactions, thus accelerating the transverse relaxation of the MRI signal (5). This property can be exploited to estimate iron content using MRI based on a reduction in T2^*^ relaxation time or an increase in R2^*^ (1/T2^*^), phase changes in susceptibility-weighted imaging (SWI), or increased susceptibility values on quantitative susceptibility mapping (QSM). Based on these techniques, iron-sensitive MRI provides a noninvasive estimation of iron content as shown in primate and postmortem studies in humans (6, 7). Recent studies using iron-sensitive MRI in PD have investigated whether iron increase in basal ganglia, particularly in the SN, can be used as a biomarker in PD diagnosis and follow-up of iron content in the disease.

Studies using iron-sensitive MRI to quantify iron content in PD have reported variable results. Some have reported increased iron content over the global SN (8–11). Others have only reported increased iron contents in some SN subregions (12, 13) or have not reported any increase in iron levels (14). Moreover, the role of iron for monitoring disease progression in PD and its correlation with clinical symptoms is still under debate (15–17). Finally, to estimate iron content, a wide variety of techniques have been employed, and no systematic comparison of the results from these studies has yet been carried out. Thus, to elucidate the current role of iron-sensitive MRI in PD and its potential application as a biomarker of PD diagnosis, we carried out a systematic review of publications employing iron-sensitive MRI to study SN in PD. We sought to determine whether MRI of iron using R2^*^, SWI, or QSM measurements could successfully distinguish PD patients from healthy controls (HCs), showing the pathological increase in iron of the SN. We also investigated the factors that could influence iron quantification and analyzed the correlations between demographic and clinical data and iron load in the SN.

## Methods

### Articles Review

The study was performed in accordance with the “Preferred Reporting Items for Systematic Reviews and Meta-analysis” (PRISMA) statement and checklist (Supplementary Table 1) (18).

To identify all the relevant literature on iron-sensitive MRI in the SN in PD, PubMed and ScienceDirect databases (from January 1994 to December 2019) were searched. A combination of the following search term was used: (“Parkinson” OR Parkinsonism OR substantia nigra) AND (“magnetic resonance imaging” OR MRI) AND (R2^*^ OR SWI OR QSM OR susceptibility). All titles and abstracts from the retrieved articles were screened, and the full text of those that could be eligible was obtained. Reference lists of identified studies were also screened for additional studies. Two independent assessors (CSM and NP) performed this literature search, selected all relevant studies based on the Patient, Intervention, Comparison, Outcome, and Study type (PICOS) guidelines, and extracted all the information on iron estimation from the selected studies (18).

Criteria for study inclusion were publication as a full-text original article redacted in English, the use of iron-sensitive MRI as a diagnostic tool (T2^*^, R2^*^, SWI, or QSM), availability of iron level estimation in the SN, and the differentiation of participants with PD from HCs. For PD, a probable diagnosis based on standard diagnostic criteria was considered sufficient for inclusion (19). Articles using additional non-iron-sensitive MRI techniques, analyzing additional regions of interest other than the SN, or investigating additional pathologies other than PD were also included. In the case of multiple publications on the same population or overlapping populations, the study describing results in the largest number of subjects was included in the meta-analysis (20, 21). Studies in the same study populations were included when they reported results in different parts of the SN or the ipsilateral and contralateral hemispheres separately. For longitudinal trials, only the data from the first period were included. The articles were included if the measurements on the level of SN were available. We did not reject articles if other techniques along with R2^*^, other regions of interest, or other pathologies along with PD were studied. Criteria for study exclusion comprised unavailability of any iron-sensitive MRI analysis (R2^*^, QSM, or SWI) in the SN or of the numerical results or absence of a HC group.

For each study, when available, the following information on the subject population was extracted: mean age of PD and HC subjects, disease duration, severity of PD [Hoehn and Yahr (HY) stage and Unified Parkinson's Disease Rating Scale (UPDRS)], and medication dose. For iron imaging, the following information was extracted: magnetic field strength, scanner vendor, number of echoes for T2^*^ measurements, type of measurements (R2^*^ or T2^*^, SWI, or QSM), and region-of-interest (ROI) location. Mean and standard deviation (SD) of the different metrics were recorded (R2^*^, SWI, and QSM). One article only provided the ranges of the values (22). Four articles did not report values as mean ± SD but as median (range) (10, 16, 23, 24).

### Statistical Meta-Analysis

Statistical parameter computation was performed using in-house software written in MATLAB R2016a software (the MathWorks, Inc., Natick, MA, USA). The meta-analysis was conducted in R version 3.5.1 (R Development Core Team, 2018) using the meta package (25). A random-effect model based on restricted maximum likelihood estimator of the between-studies variance was used.

#### Data Extraction

The mean and SDs of R2^*^, SWI, and QSM measurements in the SN were extracted from the tables or the body of the manuscript when tables were unavailable. If values were only available as part of a diagram, the values were extracted using manual measurement on an image editing tool (GIMP, version 2.8) on three separate occasions (1 day apart) and averaged.

In the studies where only the median value and range of values were available, the mean and SD were estimated as previously described (26).

To combine T2^*^ and R2^*^ values from separate studies into a single analysis, all T2^*^ values were converted to R2^*^, with the formula R2^*^ = 1/T2^*^.

#### Effect Size

Effect size was computed as the standardized mean difference (Hedge's *g*) by subtracting the mean of the HC group from that of PD patients divided by the pooled standard deviation (25). Each *g* was weighted by the inverse of its variance and adjusted for small sample bias (27). The pooled effect size was calculated separately for R2^*^, SWI, and QSM. To allow comparability within the meta-analysis, when the SN was subdivided into several ROIs and R2^*^, SWI, or QSM values for the entire SN were not available, the mean value over the SN region was calculated (10, 17, 28–33). If the mean values were separately presented for PD patients with different severity levels, they were weighted and averaged. SWI articles used different techniques, and Hedge's *g* was assumed as the absolute value of the result. An effect size of *g* > 0.70 was considered as a large effect. Confidence interval (CI) of 95% was calculated using the standard error (SE). A fixed-effect or random-effect (restricted maximum likelihood) model was used based on the *Q* statistics.

#### Between-Study Heterogeneity

The across-study heterogeneity for all the articles included in the meta-analysis was analyzed by calculating Cochrane's *Q* and *I*^2^ statistic. Values range from 0.0% (no heterogeneity) to 100% (high heterogeneity); values of 25%, 50%, and 75% have been suggested as benchmarks of low, moderate, and high heterogeneity, respectively (34).

#### Outliers

Effect sizes greater than three standard deviations from the mean were considered outliers. Results were reported with and without outliers (35).

#### Risk of Bias

The risk-of-bias analysis in individual studies was performed with a tool for the quality assessment of studies of diagnostic accuracy (QUADAS). The rating was performed by two independent raters (NP and CSM), and discordant ratings were resolved by consensus. The QUADAS questionnaire included 14 items covering the following issues: reference standard, covered patient spectrum, verification bias, disease progression bias, review bias, incorporation bias, clinical review bias, test execution, indeterminate results, and study withdrawals (Supplementary Table 2). Publication bias across studies for each outcome measure was examined by visually inspecting the funnel asymmetry plot and by applying the Egger regression intercept test.

#### Other Statistical Tests

Between-group differences; differences in R2^*^, SWI, and QSM values between SN subregions; the effect of magnetic field strength; the effect of MRI vendor; and the effect of ROI delineation methods were assessed using the nonparametric Wilcoxon, Mann–Whitney, and Kruskal–Wallis test.

#### Correlations

To assess the relationship between R2^*^, SWI, and QSM values with the clinical characteristics of patients [age, disease duration, UPDRS levels, H&Y stage, or technical parameters (number of echoes and voxel size)], a correlation analysis was performed. Correlation coefficients were computed between R2^*^, SWI, and QSM values and the clinical scores. To correct for multiple comparisons across several clinical scores, an approximate multivariate permutation test was conducted, and the sampling distribution was built to calculate the corrected *p*-value as the proportion of values that were larger than the observed correlation coefficient value (36).

## Results

The search of the database revealed 479 results in both PubMed and 1,425 ScienceDirect databases. After applying inclusion and exclusion criteria on the basis of titles and/or abstracts, 86 full-text articles were reviewed. Of these, 40 articles were excluded for the following reasons: the average R2^*^, QSM, or SWI results in the SN were not measured or not explicitly reported (*n* = 28), presence of duplicated data (*n* = 4), review articles (*n* = 8). Forty-six studies were included in the meta-analysis: 3 T2^*^ based, 24 R2^*^ based, 10 SWI, and 17 QSM-based (Supplementary Figure 1). Eight of these studies presented measurements for both R2^*^ and QSM. We show the relevant publications, population characteristics, and technical details of the included studies in Tables 1, 2 for R2^*^, Tables 4, 5 for SWI, and Tables 7, 8 for QSM.

**Table 1:** Demographic and clinical data of subjects of the articles included in the R2^*^ meta-analysis.

**References**	**Age PD**	**Disease duration PD**	**UPDRS level PD**	**H&Y PD**	**LEDD PD**	**Age HC**
Ordidge et al. (22)	47–72	2–13	N/A	1.5-3	N/A	47–72
Graham et al. (9)	61.4 (7.3)	11.1 (4.5)	N/A	N/A	N/A	64.0 (6.6)
Martin et al. (28)	61.9 (9.0)	3.2 (1.7)	16.7 (7.1)	N/A	N/A	55.9 (7.3)
Baudrexel et al. (32)	62.2 (10.2)	4.0 (2.3)	18.3 (6.1)	1.7 (0.5)	N/A	62.3 (10.8)
Péran et al. (29)	61.9 (11.1)	4.5 (2.5)	12.0 (5.9)	1.7 (0.5)	886.8 (399.5)	57.4 (9.7)
Focke et al. (37)	66.3 (7.8)	N/A	N/A	N/A	N/A	67.6 (10.5)
Du et al. (20)	60.8 (8.2)	4.2 (4.7)	23.5 (15.1)	1.8 (0.6)	528.0 (401.0)	59.8 (7.0)
Bunzeck et al. (33)	66.3 (9.0)	6.3 (4.4)	34.6 (17.4)	N/A	393.8 (339.0)	66.0 (9.1)
Rossi et al. (23)	67.5 (12.9)	1.4 (1.0)	25 (11.8)	N/A	N/A	67.0 (6.5)
Ulla et al. (13)	60.2 (10.7)	5.7 (4.4)	12.1 (8.5)	1.9 (0.7)	614.0 (317.0)	57.0 (8.5)
Lewis et al. (38)	60.6 (8.0)	4.4 (4.7)	23.8 (15.4)	1.7 (0.6)	535.0 (400.0)	59.9 (7.0)
Barbosa et al. (39)	66.0 (8.0)	8.1 (4.1)	N/A	2.3 (0.6)	N/A	64.0 (7.0)
He et al. (31)	58.0 (8.8)	2.8 (1.6)	15.6 (6.22)	1.4 (0.5)	N/A	60.5 (6.5)
Murakami et al. (40)	72.0 (7.5)	2.7 (2.3)	N/A	2.0 (0.6)	N/A	69.7 (8.6)
Pyatigorskaya et al. (8)	54.3 (10.9)	5.2 (4.2)	18.6 (9.1)	1.6 (0.6)	N/A	55.8 (7.4)
Reimão et al. (26)	65.1 (9.2)	1-5	27.4 (12.8)	2.0 (0.0)	N/A	61.2 (7.3)
Wieler et al. (41)	59.8 (7.3)	1.8 (1.3)	14.3 (5.1)	N/A	N/A	56.0 (6.9)
Guan et al. (30)	55.4 (9.5)	4.7 (3.8)	27.1 (15.4)	2.2 (0.7)	N/A	56.6 (9.9)
Hopes et al. (10)	60.4 (3.2)	5.1 (0.6)	28.8 (2.6)	1.9 (0.2)	N/A	60.0 (2.4)
Isaias et al. (12)	62.8 (9.0)	7.5 (3.57)	14.5 (5.78)	2	502.0 (183.0)	60 (8.74)
Langkammer et al. (42)	64.7 (8.8)	3.4	31.3 (14.6)	2 (0.5)	182.5 (436.9)	65 59.3)
Du et al. (43)	66.3 (9.5)	4.5 (4.5)	21.5 (14.7)	1.7 (0.7)	669.0 (464.0)	66.2 (10.2)
	64.5 (9.2)	4.3 (4.1)	19.8 (5.9)	N/A	N/A	63 (9)
Langley et al. (13)	63.6 (7.0)	6.1 (4.6)	22.2 (12.4)	N/A	N/A	63.1 (7.2)
Pesch et al. (16)	59.5 (3.5)	4.9 (1.5)	34 (10.1)	N/A	N/A	65.5 (4.7)
Ghassaban et al. (44)	61.8 (6.4)	N/A	N/A	N/A	N/A	63.4 (8)
Arribarat et al. (11)	65.2 (6.6)	6.8 (4.7)	11.4 (4.9)	N/A	N/A	66 (4.9)
Li et al. (14)	68.2 (6.1)	N/A	27 (15.7)	N/A	N/A	64.8 (8)

*Data are presented as mean (STD). N/A, not available*.

**Table 2:** Technical characteristics of the studies included in the R2^*^ meta-analysis.

**References**	**Magnetic field**	**MRI machine**	**Method**	**ROIs drew in**	**No. echoes**	**Voxel size**
Ordidge et al. (22)	3 T	Magnex Scientific Ltd.	T2*	T2* weighted	6	N/A
Graham et al. (9)	1.5 T	Marconi Medical Systems	R2*	T2 weighted	6	0.9 × 0.9 × 2.5
Martin et al. (28)	3 T	Magnex Scientific Ltd.	R2*	T2* weighted	6	2.3 × 2.3 × 5.0
Baudrexel et al. (32)	3 T	Siemens	T2*	T1 map	8	1.0 × 1.0 × 2.0
Péran et al. (29)	3 T	Siemens	R2*	T2* weighted	6	1.8 × 1.8 × 1.8
Focke et al. (37)	3 T	Siemens	R2*	T1 MPRAGE + MT	5	1.7 × 1.7 × 1.7
Du et al. (20)	3 T	Siemens	R2*	T2 weighted	6	1.0 × 1.0 × 1.0
Bunzeck et al. (33)	3 T	Siemens	R2*	MT	6	1.0 × 1.0 × 1.0
Rossi et al. (23)	3 T	Siemens	R2*	T2* map	5	0.6 × 0.6 × 4.0
Ulla et al. (13)	1.5 T	Siemens	R2*	T2* weighted	6	2.2 × 2.2 × 2.5
Lewis et al. (38)	3 T	Siemens	R2*	T2 weighted	6	1.0 × 1.0 × 1.0
Barbosa et al. (39)	3 T	Philips	R2*	QSM	4	0.5 × 0.5 × 2.0
He et al. (31)	3 T	GE	R2*	QSM	8	0.5 × 0.5 × 2.0
Murakami et al. (40)	3 T	GE	R2*	R2* map	11	1.5 × 2.5 × 1.5
Pyatigorskaya et al. (8)	3 T	Siemens	R2*	T2 weighted	6	2.0 × 2.0 × 2.0
Reimão et al. (26)	3 T	Philips	T2*	Spin-echo T1 neuromelanin sensitive	7	1.2 × 0.9 × 4.0
Wieler et al. (41)	3 T	Magnex Scientific Ltd.	R2*	T2* weighted	6	2.3 × 2.3 × 5.0
Guan et al. (30)	3 T	GE	R2*	QSM	8	0.8 × 0.8 × 2.8
Hopes et al. (10)	3 T	Philips	R2*	T2* weighted	15	2.0 × 2.0 × 2.0
Isaias et al. (12)	3 T	Philips	R2*	T2* weighted	2	1 × 1 × 1
Langkammer et al. (42)	3 T	Siemens	R2*	QSM	6	0.9 × 0.9 × 2.0
Du et al. (21)	3 T	Siemens	R2*	T2 weighted	8	0.9 × 0.9 × 2.0
Langley et al. (13)	3 T	Siemens	R2*	T2* weighted	6	0.5 × 0.5 × 1
Pesch et al. (16)	3 T	Philips	R2*	3D T1 weighted	N/A	1.5 × 1.5 × 1.5
Ghassaban et al. (44)	3 T	GE	R2*	QSM	N/A	0.86 × 0.86 × 1
Arribarat et al. (11)	3 T	Siemens	R2*	T2 weighted	6	1.8 × 1.8 × 1.8
Li et al. (14)	3 T	Siemens	R2*	QSM	8	0.63 × 0.63 × 2.0

No significant publication bias was identified by a funnel plot and Egger regression intercept test. The funnel plots were symmetrical, and the Egger regression intercept test had no significant publication bias for the meta-analysis of R2^*^, SWI, and QSM changes (*p* = 0.13, *p* = 0.58, and *p* = 0.45, respectively, Supplementary Figures 2–4). The risk-of-bias analysis in individual studies is presented in Supplementary Table 2.

### Regions of Interest in the SN

All included studies investigated differences in R2^*^/T2^*^, QSM, or SWI in the SN. Mean and SDs of R2^*^ or T2^*^, SWI, and QSM were extracted for each ROI delineated in each study. In all the studies, SN ROIs were either manually drawn or calculated by using semiautomatic methods followed by manual correction. In all studies, one or two reviewers blinded to the subject characteristics analyzed the MRI images and drew the contours of the SN (or its different subregions) by hand. As for studies using R2^*^ MRI, 16 studies drew the SN ROI on anatomical images [T2-weighted images (8, 9, 11, 20, 38, 43), T2^*^-weighted images (10, 12, 13, 17, 22, 28, 29, 41), or T1-weighted (16, 24)], and nine used quantitative maps [six used QSM maps (14, 30, 31, 39, 42, 44), one used an R2^*^ map (40), one used a T2^*^ map (23), one used a T1 map (32), two used magnetization transfer (MT) images, and one of them combined with an anatomical T1-weighted image (33, 37)]. The majority of studies assumed that the SN corresponded to the hypointense region on T2-weighted images between the red nucleus and the cerebral peduncle (8, 10, 20, 23, 38). For SWI (12, 23, 45–52) and QSM studies (14, 30, 31, 39, 40, 42–44, 53–61), all ROIs were manually segmented on the phase or QSM images.

Some studies placed ROIs in different subregions of the SN. In the R2^*^-based studies, some divided the SN into SNc and SN pars reticulata (SNr) (17, 28, 30, 39, 47, 51), defining the SNr as a hyperintense or hypointense region (depending on the image contrast) in the ventrolateral midbrain and the SNc as the region between the SNr and red nucleus (28, 30, 41). In three studies, rostral and caudal SNs were defined as the upper and lower parts of the SN (20, 32). Also, several articles studied the differences between the SN contralateral (opposite side) or ipsilateral (same side) to the most affected limb of the body (20, 31, 32, 49, 53). Three others analyzed the lateral, central, or medial of the SN separately. As for the SWI-based studies, two divided the SN into SNc and SNr (47, 51), two in ipsilateral and contralateral SNs (12, 49), and one divided the SNc into lateral and medial (23). As for the QSM-based studies, three measured the magnetic susceptibility in the ipsilateral and contralateral SNs (31, 53, 57), seven in SNc and SNr (30, 39, 43, 54, 56–58), and two in the anterior and posterior SNs (53, 55). The subregion segmentations were mostly performed on QSM images, while one study also used neuromelanin-sensitive imaging to help the SNc delineation (56).

### Group Comparisons and Effect Sizes

#### R2^*^ Meta-Analysis

**Figure 1:**
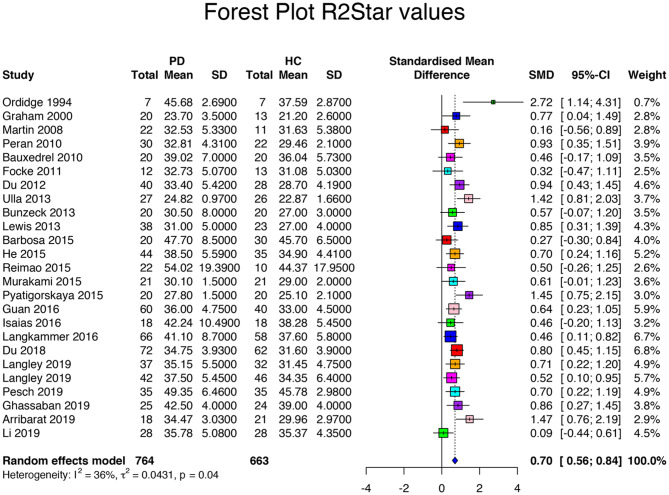
Forest plot of significant R2* values of the 26 articles included in the meta-analysis. Forest plot of the computed disease effect sizes (Hedge's g, x-axis) of studies included into the meta-analysis on R2* measures of the substantia nigra when comparing PD patients and controls. Pooled SMD (95%) (0.7, [0.56, 0.84]) is denoted by a blue diamond.

**Figure 2:**
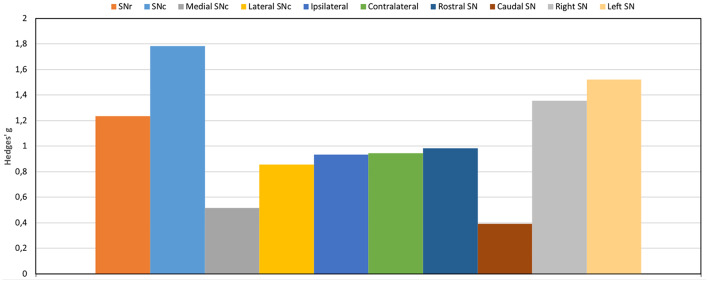
Graphical representation of the different effect sizes in each division of the SN ROIs using R2*.

Searching the database returned 27 R2^*^/T2^*^-based articles. The R2^*^ meta-analysis included a population of 1,629 subjects with 879 PD patients and 750 HCs. Among all studies, the mean age of the patients (62.8 ± 3.7 years, range 54 to 72 years) did not differ from that of the HCs (61.0 ± 4.3 years, *p* = 0.35). Disease duration was 5.3 ± 2.2 (range 1.4 to 11.1 years). UPDRS scores were 21.9 ± 7.8 (range 12.0 to 34.6). HY score was 1.9 ± 0.3 (range 1.4 to 2.6). The levodopa-equivalent daily dose (LEDD) was specified in eight articles, with a mean of 539.4 ± 204.6 mg (Table 1). The between-study variation was *I*^2^ = 78%, which indicated a relatively high heterogeneity between studies. For R2^*^ measurements, the standardized mean difference was 0.84 with a CI of 95% between 0.60 and 1.08 (range: 0.16 to 4.36, *p* < 0.001, Table 3, Supplementary Figure 5). R2^*^ ranged from 23.70 to 54.02 s^−1^ (mean 36.88 ± 7.51 s^−1^) for PD patients and from 21.20 to 45.78 s^−1^ (mean 33.28 ± 6.44 s^−1^) for HCs (*p* = 0.03). One study was an outlier with effect sizes greater than three standard deviations (10). After the outlier was excluded, the heterogeneity was *I*^2^ = 36%, indicating that moderate heterogeneity and standardized mean difference was 0.70 with a CI of 95% between 0.56 and 0.84 (range: 0.16 and 2.72, *p* = 0.04, Figure 1). R2^*^ ranged from 23.70 to 54.02 s^−1^ (mean 36.63 ± 7.44 s^−1^) for PD patients and from 21.20 to 45.78 s^−1^ (mean 33.12 ± 6.52 s^−1^) for HCs (*p* = 0.04). All studies have shown a relative increase in R2^*^ values in PD. Differences existed in R2^*^ values between studies that used anatomical images (T1 or T2 weighted) to draw the ROIs or QSM maps. Anatomical-based ROI had lower R2^*^ values (mean 33.3 ± 8.9 s^−1^) than QSM-based ROI (mean 38.1 ± 7.3 s^−1^, *p* = 0.33) and higher Hedge's *g* (1.3 ± 1.2 vs. 0.6 ± 0.2, *p* = 0.07), but these differences were not significant. As for the SN subregions, increased iron contents were observed at the level of the SNc in PD, while there were no significant changes in iron content in the SNr (*p* = 0.003 vs. *p* = 0.07), with effect size significantly higher in the SNc compared to the SNr (1.78 vs. 1.23, respectively, *p* = 0.09). R2^*^ was increased in the lateral compared to medial SNc (*p* = 0.02 and 0.06, respectively) with nonsignificantly higher effect size in lateral than in median SNc (0.86 vs. 0.52, respectively, *p* = 0.11) possibly due to the low number of articles reporting distinct measurements, while both ipsilateral and contralateral sides had the same effect size (0.95 vs. 0.93, *p* = 0.89) with no difference for R2^*^ (*p* = 0.31) (Figure 2).

**Table 3:** Meta-analysis results: SWI mean values of PD and HC were used to calculate the effect size value and confidence interval.

**References**	**SN division**	**PD**			**HC**			**Hedge's *g***	**SE**	**Confidence interval**	
		**N**	**Mean**	**SD**	**N**	**Mean**	**SD**			**Low**	**High**
Ordidge et al. (22)	Global^NA^	7	45.68	2.69	7	37.59	2.87	2.72	0.77	1.22	4.23
Graham et al. (9)	Global^*^	20	23.70	3.50	13	21.20	2.60	0.76	0.37	0.05	1.48
Martin et al. (28)	SNc lateral^**^	22	26.90	2.80	11	22.80	2.80	1.43	0.41	0.62	2.23
	SNc medial^*^		29.00	7.20		25.90	7.30	0.42	0.37	−0.31	1.15
	SNr lateral		35.80	5.50		37.90	5.50	−0.37	0.37	−1.10	0.36
	SNr medial		38.40	5.80		39.90	5.90	−0.25	0.37	−0.98	0.48
Baudrexel et al. (32)	Rostral ipsilateral^*^	20	27.17	3.14	20	24.45	2.28	0.97	0.34	0.31	1.63
	Rostral contralateral^*^		28.01	3.86		25.32	2.64	0.80	0.34	0.15	1.44
	Caudal ipsilateral		19.60	10.40		21.65	10.10	−0.20	0.33	−0.82	0.43
	Caudal contralateral		20.04	10.60		20.75	7.90	−0.07	0.32	−0.69	0.55
Péran et al. (29)	Right^***^	30	33.34	4.72	22	29.89	2.08	0.90	0.30	0.31	1.46
	Left^***^		32.27	3.90		29.02	2.11	0.99	0.30	0.40	1.56
Focke et al. (37)	Right	12	32.42	5.58	13	31.31	5.15	0.20	0.40	−0.59	0.99
	Left		33.03	4.30		30.20	4.30	0.64	0.41	−0.17	1.44
Du et al. (20)	Global^***^	40	33.40	5.42	28	28.70	4.19	0.94	0.26	0.43	1.45
Ulla et al. (13)	SNc^**^	27	22.58	0.68	26	20.65	0.60	2.96	0.40	2.18	3.75
	SNr^*^		27.07	1.26		25.09	0.92	1.76	0.34	1.13	2.40
Rossi et al. (23)	Medial SNc^***^	37	51.00	10.00	21	43.00	7.00	0.87	0.29	0.31	1.43
	Lateral SNc^***^		50.00	10.00		42.00	6.00	0.90	0.29	0.34	1.46
Bunzeck et al. (33)	Left	20	30.00	8.00	20	26.00	3.00	0.65	0.33	0.01	1.29
	Right		31.00	8.00		28.00	3.00	0.49	0.32	−0.14	1.12
Lewis et al. (38)	Global^**^	38	31.00	5.00	23	27.00	4.00	0.85	0.28	0.31	1.39
Barbosa et al. (39)	SNc^*^	20	52.80	11.70	30	47.70	8.40	0.51	0.32	−0.06	1.09
	Global^*^		47.70	8.50		45.70	6.50	0, 27	0.29	−0.30	0.84
He et al. (31)	Contralateral^*^	44	38.90	5.91	35	34.90	4.41	0.75	0.24	0.29	1.21
	Ipsilateral		38.10	5.26		34.90	4.41	0.65	0.23	0.19	1.10
Reimão et al. (26)	Global	22	54.02	19.39	10	44.37	17.95	0.50	0.39	−0.26	1.25
	Lateral		46.60	19.28		35.45	7.58	0.65	0.39	−0.11	1.42
	Central		50.07	22.47		40.97	10.46	0.45	0.39	−0.30	1.21
	Medial		58.27	15.96		49.77	17.73	0.50	0.39	−0.26	1.26
Murakami et al. (40)	Global^*^	21	30.10	1.50	21	29.00	2.00	0.61	0.32	−0.01	1.23
Pyatigorskaya et al. (8)	Global^*^	20	27.80	1.50	20	25.10	2.10	1.45	0.36	0.75	2.15
Wieler et al. (41)	Lateral SNc^*^	19	26.23	3.08	13	22.82	5.13	0.83	0.38	0.09	1.56
Guan et al. (30)	SNc^*^	60	28.18	3.57	40	25.25	3.03	0.86	0.22	0.45	1.28
	SNr		43.79	5.28		40.94	5.71	0.52	0.21	0.11	0.93
Hopes et al. (10)	Left^*^	70	46.44	2.17	20	37.60	0.87	4.46	0.42	3.64	5.29
	Right^*^		46.22	2.31		36.90	1.16	4.37	0.42	3.56	5.18
Isaias et al. (12)	Ipsilateral	18	41.17	10.96	18	37.47	5.16	0.42	0.34	−0.24	1.08
	Contralateral		43.30	10.01		39.09	5.74	0.50	0.34	−0.16	1.17
Langkammer et al. (42)	Global^*^	66	41.10	8.70	58	37.60	5.80	0.46	0.18	0.11	0.82
Du et al. (43)	SNc^***^	72	28.00	0.40	62	25.90	0.40	5.22	0.37	4.51	5.93
	SNr^**^		41.50	1.00		37.30	1.10	3.98	0.30	3.40	4.58
Langley et al. (13)	SNc cohort 1^***^		32.50	5.60	32	27.50	4.30	0.98	0.26	0.48	1.48
	SNr cohort 1^*^		37.80	5.40	32	35.40	5.20	0.44	0.25	−0.03	0.93
	SNc cohort 2^***^	42	34.30	4.90	46	29.50	4.40	1.02	0.23	0.58	1.47
	SNr cohort 2		40.70	6.00		39.20	8.40	0.20	0.21	−0.22	0.62
Pesch et al. (16)	Global	35	49.35	6.46	35	45.78	2.98	0.71	0.25	0.22	1.18
Ghassaban et al. (44)	Right^*^	25	42.00	4.00	24	39.00	4.00	0.74	0.30	0.16	1.32
	Left^*^	25	43.00	4.00		39.00	4.00	0.98	0.31	0.39	1.58
Arribarat et al. (11)	Posterior	18	25.96	3.12	21	25.02	2, 14	0.35	0.33	−0.29	0.98
	Anterior^**^		31.05	3.61		27.26	2.97	1.13	0.35	0.45	1.81
	Global^***^		34.47	3.03		29.96	2.97	1.47	0.36	0.76	2.19
Li et al. (14)	Global	28	35.78	5.08	28	35.37	4.35	0.09	0.27	−0.44	0.61

*All SN subdivisions are included*.

* *Significantly different from controls (p < 0.05)*.

** *Significantly different from controls (p < 0.005)*.

*** *Significantly different from controls (p < 0.001). NA, non available*.

#### SWI Meta-Analysis

**Figure 3:**
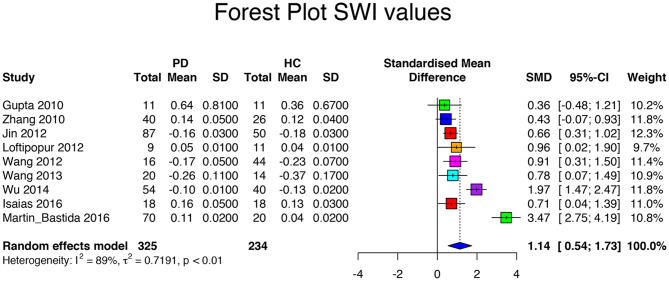
Forest plot of significant SWI values of the 10 articles included in the meta-analysis. Forest plot of the computed disease effect sizes (Hedge's g, x-axis) of studies included into the meta-analysis on QSM measures of the substantia nigra when comparing PD patients and controls. Pooled SMD (95%) (1.14, [0.54, 1.73]) is denoted by a blue diamond.

Searching the database returned 10 SWI-based articles. The SWI meta-analysis included a population of 655 subjects with 361 PD patients and 294 HCs (Table 4). Among all studies, the mean age of the patients (62.2 ± 3.6 years, range 56 to 67 years) did not differ from that of the HCs (59.9 ± 4.7 years, *p* = 0.17). Disease duration was 4.3 ± 2.3 (range 1.4 to 8.1 years). UPDRS scores were 20.2 ± 6.9 (range 14.8 to 31.5). HY score was 2.1 ± 0.4 (range 1.8 to 2.7). The LEDD was specified in two articles only, with a mean of 532.5 ± 43.13 mg. The between-study variation of SWI values was *I*^2^ = 89%, which indicated a high heterogeneity between studies. Most SWI-based studies calculated the phase of images, but others used the relative susceptibility, the SWI contrast, or the SWI hypointensity (Table 5); consequently, Hedge's *g* was calculated as the absolute value. In the SWI-based studies, the standardized mean difference was 1.14 with a CI of 95% between 0.54 and 1.73 (range 0.36 to 3.47), confirming the difference in susceptibility values between HC and PD patients (Table 6, Figure 3). There were not enough SWI-based articles to access the quality of SWI-based discrimination of different SN subregions, contralateral or ipsilateral SN sides, or field strengths.

**Table 4:** Demographic and clinical data of subjects of the articles included in the SWI meta-analysis.

**References**	**PD**					**HC**
	**Age**	**Disease duration**	**UPDRS**	**H/Y**	**LEDD (mg)**	**Age**
Gupta et al. (45)	61.5 (5.9)	8.1 (3.9)	21.4 (14.6)	N/A	N/A	54.9 (3.1)
Zhang et al. (49)	58.7 (12.8)	3.6 (2.9)	19.0 (7.8)	N/A	N/A	57.3 (11.6)
Jin et al. (50)	59.8 (11.1)	3.1 (3.0)	14.8 (9.2)	1.75 (0.8)	N/A	57.36 (13.4)
Lotfipour et al. (51)	64.7 (13.3)	N/A	N/A	1.8 (0.83)	N/A	59.2 (8.6)
Wang et al. (46)	63.3 (10.6)	2.5 (1.7)	N/A	N/A	N/A	59.4 (11.8)
Rossi et al. (23)	67.5 (12.9)	1.4 (1.0)	25 (11.8)	N/A	N/A	67.0 (6.5)
Wang et al. (47)	67.7 (9.3)	3.0 (2.7)	N/A	13 < 2.5	N/A	64.3 (12.7)
				7 > 3		
Wu et al. (48)	65.6 (5.8)	N/A	N/A	2.58 (1.29)	N/A	66.5 (6.0)
Isaias et al. (12)	62.8 (9.0)	7.5 (3.57)	14.5 (5.78)	2	502 (183)	60 (8.7)
Martin-Bastida et al. (52)	55.8 (7.2)	5.4 (2.5)	31.5 (11.6)	1.9 (0.5)	563 (344)	53.1 (11.7)

*Data are presented as mean (STD). N/A, not available*.

**Table 5:** Technical characteristics of the studies included in the SWI meta-analysis.

**References**	**Technical aspects**				
	**Magnetic field**	**Vendor**	**Method**	**ROIs drawn in**	**Voxel size (mm3)**
Gupta et al. (45)	1.5 T	Siemens	Hypointensity scores	SWI	N/A
Zhang et al. (49)	3 T	Siemens	Phase shift	Phase images	0.5 × 0.5
Jin et al. (50)	3 T	GE	Phase values	Phase images	0.8 × 0.9 × 2
Lotfipour et al. (51)	7 T	Philips	Relative susceptibility	Modulus images and then applied to SWI	Protocol A: 0.7 × 0.7 × 0.7
					Protocol B 0.4 × 0.4 × 0.5
Wang et al. (46)	1.5 T	Siemens	Phase shift	Phase images	0.5 × 1 × 2
Rossi et al. (23)	3 T	Siemens	SWI contrast	Phase images	0.9 × 0.9 × 1.5
Wang et al. (47)	3 T	GE	Phase values	Phase images	0.5 × 0.8 × 2
Wu et al. (48)	3 T	Philips	Phase values	Phase images	0.5 × 0.6 × 1.5
Isaias et al. (12)	3 T	Philips	Phase values	Phase images	1 × 1 × 1
Martin-Bastida et al. (52)	3 T	Siemens	Phase shift	Phase images	N/A

**Table 6:** Meta-analysis results: SWI mean values of PD and HC were used to calculate the effect size value and confidence interval.

**References**	**SN division**	**Method**	**PD**			**HC**			**Hedge's *g***	**SE**	**Confidence interval**	
			***N***	**Mean**	**SD**	***N***	**Mean**	**SD**			**Low**	**High**
Gupta et al. (45)	Global	SWI hypointensity	11	0.36	0.81	11	0.64	0.67	0.36	0.43	−0.48	1.21
Zhang et al. (49)	Ipsilateral	Phase values	40	0.13	0.05	26	0.12	0.04	0.21	0.25	−0.28	0.71
	Contralateral^***^			0.16	0.05		0.12	0.04	0.85	0.26	0.34	1.37
Jin et al. (50)	Global^***^	Phase values	87	−0.18	0.03	50	−0.16	0.03	0.66	0.18	0.31	1.02
Lotfipour et al. (51)	SN	Relative susceptibility	9	0.05	0.01	11	0.04	0.01	0.96	0.48	0.02	1.89
	SNc^*^			0.06	0.01		0.04	0.01	1.92	0.55	0.84	2.99
Wang et al. (46)	Global^*^	Phase values	16	−0.23	0.05	44	−0.17	0.07	0.91	0.30	0.31	1.50
Rossi et al. (23)	Lateral SNc	SWI contrast	36	7.20	3.50	21	9.10	5.20	0.45	0.28	−0.10	0.99
	Medial SNc^*^			5.70	5.90		9.90	6.50	0.68	0.28	0.12	1.23
Wang et al. (47)	SNc^**^	Phase values	20	0.03	0.03	14	0.08	0.02	1.85	0.42	1.03	2.67
	SNr^**^			−0.55	0.18		−0.37	0.17	1.00	0.37	0.27	1.72
Wu et al. (48)	Global^*^	Phase values	54	−0.13	0.01	40	−0.10	0.02	1.97	0.25	1.48	2.47
Isaias et al. (12)	Ipsilateral	Phase values	18	0.16	0.05	18	0.13	0.03	0.71	0.34	0.04	1.39
	Contralateral			0.15	0.05		0.13	0.03	0.47	0.34	−0.19	1.14
Martin-Bastida et al. (52)	Global^**^	Phase values	70	0.11	0.02	20	0.04	0.02	3.47	0.36	2.76	4.18

*All SN subdivisions were included*.

*** *Significantly different from controls (p < 0.001)*.

** *Significantly different from controls (p < 0.01)*.

* *Significantly different from controls (p < 0.05)*.

#### QSM Meta-Analysis

**Figure 4:**
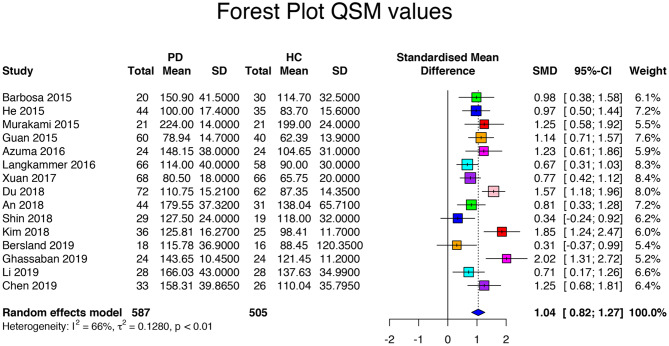
Forest plot of significant QSM values of the 16 articles included in the meta-analysis. Forest plot of the computed disease effect sizes (Hedge's g, x-axis) of studies included into the meta-analysis on QSM measures of the substantia nigra when comparing PD patients and controls. Pooled SMD (95%) (1.04, [0.82, 1.27]) is denoted by a blue diamond.

The database search returned 17 QSM-based articles. The QSM meta-analysis included a population of 1,154 subjects with 652 PD patients and 502 HC. Among all studies, the mean age of the patients (64.4 ± 5.5 years, range 50 to 72 years) did not differ from that of the HC (63.4 ± 4.3 years, *p* = 0.55). Disease duration was 4.2 ± 1.9 (range 1 to 8.1 years). UPDRS scores were 24.4 ± 10.1 (range 13.0 to 39.5). HY score was 2.1 ± 0.4 (range 1.4 to 2.8). The LEDD was specified in three articles, with a mean of 539.4 ± 204.6 mg (Tables 7, 8). The between-study variation was *I*^2^ = 75% which indicated a relatively high heterogeneity between studies. The standard mean difference was 1.13 with a CI of 95% between 0.86 and 1.39 (range = 0.34 to 3.83). The QSM values for PD patients ranged from 78.9 to 239.0 ppb (mean 138.8 ± 44.3 ppb) and for HC from 62.4 to 199.0 ppb (mean 112.9 ± 37.7 ppb, *p* = 0.001, Table 9, Supplementary Figure 6). One study was an outlier (61). After the outlier was excluded, the heterogeneity was *I*^2^ = 66%, indicating moderate heterogeneity and a standardized mean difference of 1.04 with a CI of 95% between 0.82 and 1.27 (range: 0.34 and 2.02, *p* < 0.01, Figure 4). The QSM values ranged from 78.9 to 224.0 ppb (mean 138.8 ± 44.3 ppb) for PD patients and from 62.39 to 199.0 ppb (mean 112.9 ± 37.7 ppb, *p* = 0.001) for HC. As for SN subregions, increased QSM values were observed at both levels of the SNc and the SNr in PD (*p* = 0.006 vs. *p* = 0.04, respectively). Hedge's *g* was not significantly higher in the SNc than in the SNr (1.24 vs. 0.94, respectively, *p* = 0.23). Also, there was no significant difference in effect size between the ipsilateral and contralateral SNs to the most affected side (0.64 vs. 0.68, *p* = 0.71) and no significant difference in QSM values between the ipsilateral and contralateral SNs (*p* = 0.22).

**Table 7:** Demographic and clinical data of subjects of the articles included in the QSM meta-analysis.

**References**	**PD**					**HC**
	**Age**	**Disease duration**	**UPDRS level**	**H&Y stage**	**LEDD (mg)**	**Age**
Barbosa et al. (39)	66 (8.0)	8.1 (4.1)	N/A	2.3 (0.6)	N/A	64.0 (7.0)
He et al. (31)	58 (8.8)	2.8 (1.6)	15.57 (66.22)	1.4 (0.5)	N/A	60.5 (6.5)
Murakami et al. (40)	72.0 (7.5)	2.7 (2.3)	N/A	2.0 (0.6)	N/A	69.7 (8.6)
Azuma et al. (53)	63.3 (11.0)	6.4 (3.6)	20.8 (11.6)	N/A	456.2 (265.2)	64.1 (10.0)
Guan et al. (30)	58.5 (7.5)	5.7 (4.2)	33.65 (12.4)	2.6 (2.5)	N/A	56.6 (9.9)
Langkammer et al. (42)	60.1 (6.2)	6.2 (4.2)	18.6 (7.6)	2 (1)	N/A	58.1 (8.7)
Xuan et al. (54)	67.3 (9.9)	4.1 (2.1)	13 (7.1)	2.1 (0.95)	N/A	66.9 (9.1)
Du et al. (21)	70.6 (9)	1.5 (8.5)	20.3 (10.7)	1.9 (0.7)	175 (215)	67.6 (5)
Kim et al. (60)	71.2 (6.9)	5.4 (2)	13.1 (7.2)	1.9 (0.4)	N/A	67.1 (4.7)
An et al. (59)	61.8 (6.4)	N/A	N/A	N/A	N/A	63.4 (8)
Takahashi et al. (56)	68.2 (6.1)	N/A	27 (15.7)	N/A	N/A	64.8 (8)
Shin et al. (25)	64.6 (11.2)	2.8 (2.4)	N/A	2.8 (2.4)	N/A	62.6 (10.6)
Bergsland et al. (55)	66.2 (8.5)	N/A	N/A	2.1 (0.5)	N/A	64.9 (9.2)
Shahmaei et al. (61)	60.1 (6.2)	6.2 (4.2)	18.6 (7.6)	2 (1)	N/A	58.1 (8.7)
Ghassaban et al. (44)	67.3 (9.9)	4.1 (2.1)	13 (7.1)	2.1 (0.95)	N/A	66.9 (9.1)
Li et al. (14)	71.2 (6.9)	5.4 (2)	13.1 (7.2)	1.9 (0.4)	N/A	67.1 (4.7)
Chen et al. (58)	70.6 (9)	1.5 (8.5)	20.3 (10.7)	1.9 (0.7)	175 (215)	67.6 (5)

*Data are presented as mean (STD). N/A, not available*.

**Table 8:** Technical characteristics of the studies included in the QSM meta-analysis.

**References**	**Magnetic field**	**Vendor**	**Values obtained**	**ROIs drawn in**	**Voxel size**
Barbosa et al. (23)	3 T	Philips	Susceptibility	QSM	0.5 × 0.5 × 2.0
He et al. (31)	3 T	GE	Susceptibility	QSM	0.5 × 0.5 × 2.0
Murakami et al. (40)	3 T	GE	Susceptibility	QSM	1.5 × 2.5 × 1.5
Guan et al. (30)	3 T	GE	Susceptibility	QSM	0.8 × 0.8 × 2.8
Azuma et al. (53)	3 T	Siemens	Susceptibility	QSM	0.9 × 0.9 × 2.0
Langkammer et al. (42)	3 T	Siemens	Susceptibility	QSM	0.9 × 0.9 × 2.0
Xuan et al. (54)	3 T	GE	Susceptibility	QSM	0.6 × 0.6 × 2.8
Du et al. (21)	3 T	Siemens	Susceptibility	QSM	0.9 × 0.9 × 2.0
Kim et al. (60)	3 T	Siemens	Susceptibility	QSM	1 × 1 × 1
An et al. (59)	3 T	Siemens	Susceptibility	QSM	0.62 × 0.62 × 2
Takahashi et al. (56)	3 T	GE	Susceptibility	QSM	0.57 × 0.86 × 2.4
Shin et al. (25)	3 T	Philips	Susceptibility	QSM	0.4 × 0.4 × 2
Bergsland et al. (55)	3 T	GE	Susceptibility	QSM	0.5 × 1 × 2
Shahmaei et al. (61)	3 T	Siemens	Susceptibility	QSM	1 × 1 × 1.5
Ghassaban et al. (44)	3 T	GE	Susceptibility	QSM	0.86 × 0.86 × 1
Li et al. (14)	3 T	Siemens	Susceptibility	QSM	0.63 × 0.63 × 2.0
Chen et al. (58)	3 T	Philips	Susceptibility	QSM	0.5 × 0.5 × 2

**Table 9:** Meta-analysis results: QSM mean values of PD and HC are used to calculate the effect size value and confidence interval.

**References**	**SN division**	**PD**			**HC**			**Hedge's *g***	**SE**	**Confidence interval**	
		***N***	**Mean**	**SD**	***N***	**Mean**	**SD**			**Low**	**High**
Barbosa et al. (23)	SN^**^	20	150.90	41.50	30	114.70	32.50	0.98	0.31	0.38	1.58
	SNc^***^		186.70	53.20		140.10	38.50	1.02	0.31	0.42	1.62
He et al. (31)	Ipsilateral^***^	44	100.00	16.50	35	83.70	15.60	1.00	0.24	0.53	1.47
	Contralateral^***^		100.00	18.30		83.70	15.60	0.94	0.24	0.47	1.41
Murakami et al. (40)	Global^*^	21	224.00	14.00	21	199.00	24.00	1.25	0.34	0.59	1.91
Azuma et al. (53)	Global^**^	24	148.15	45.75	24	104.70	31.00	1.09	0.31	0.49	1.70
	Contralateral aSN^***^		186.6	57.50		142.90	43.70	0.84	0.30	0.25	1.43
	Contralateral mSN^***^		166.6	53.20		104.60	28.70	1.43	0.32	0.79	2.06
	Contralateral pSN^***^		120.8	38.70		66.50	25.60	1.63	0.33	0.97	2.28
	Ipsilateral aSN		178.60	54.70		142.90	43.70	0.71	0.30	0.13	1.29
	Ipsilateral mSN^*^		141.40	51.10		104.60	28.70	0.87	0.30	0.28	1.47
	Ipsilateral pSN^*^		94.70	38.00		66.50	25.60	0.86	0.30	0.26	1.45
Guan et al. (30)	SNc^**^	60	47.00	15.00	40	20.00	15.00	1.79	0.24	1.31	2.26
	SNr		122.50	25.00		101.00	25.00	0.85	0.21	0.44	1.27
Langkammer et al. (42)	Global^***^	66	114.00	40.00	58	90.00	30.00	0.67	0.18	0.31	1.03
Xuan et al. (54)	SNc (young)^**^	35	37.00	25.00	24	25.00	15.00	0.55	0.27	0.02	1.08
	SNr (young)^*^		117.00	20.00		104.00	22.00	0.62	0.27	0.08	1.15
	SNc (old)^**^	33	45.00	18.00	22	28.00	20.00	0.89	0.29	0.33	1.46
	SNr (old)^*^		123.00	27.00		106.00	30.00	0.59	0.28	0.04	1.14
Du et al. (43)	SNc^**^	72	64.20	15.21	62	44.80	14.35	1.30	0.19	0.93	1.68
	SNr^**^		157.30	15.21		129.90	14.35	1.84	0.21	1.43	2.24
Kim et al. (60)	Global^***^	36	125.81	16.27	25	98.41	11.70	1.85	0.31	1.25	2.46
An et al. (59)	Global^**^	44	179.55	65.72	31	138.04	37.32	0.74	0.24	0.26	1.21
Takahashi et al. (56)	SNc^*^	18	87.67	24.48	18	67.78	24.48	0.79	0.35	0.11	1.47
	SNc dorsolateral^*^		85.78	28.45		52.19	23.01	1.27	0.37	0.55	1.99
Shin et al. (25)	SNc ipsilateral	29	123.00	39.00	19	121.00	32.00	0.05	0.30	−0.52	0.63
	SNc contralateral		132.00	39.00		121.00	32.00	0.30	0.30	−0.28	0.88
	SNr ipsilateral		125.00	39.00		115.00	39.00	0.25	0.30	−0.33	0.83
	SNr contralateral		130.00	40.00		115.00	39.00	0.37	0.30	−0.21	0.96
Bergsland et al. (55)	Ventral posterior^***^	32	113.10	36.20		62.10	29.10	1.51	0.39	0.74	2.27
	Ventral anterior^*^		160.70	40.40		124.00	38.40	0.91	0.36	0.20	1.62
	Dorsal posterior		78.00	35.00		66.90	23.50	0.36	0.35	−0.32	1.04
	Dorsal anterior		111.30	36.00		100.80	390.40	0.04	0.34	−0.64	0.71
Shahmaei et al. (61)	Global^***^	15	239.00	21.00	15	146.00	26.00	3.83	0.63	2.60	5.06
Ghassaban et al. (44)	Right^*^	24	139.80	10.40	24	115.40	11.60	2.18	0.37	1.46	2.90
	Left^*^		147.50	10.50		127.50	10.80	1.85	0.35	1.17	2.53
Li et al. (14)	Global^*^	28	166.03	43,00	28	137.63	34.99	0.71	0.28	0.17	1.26
Chen et al. (58)	SNc^*^	33	163.47	49.16	26	85.18	30.57	1.84	0.31	1.22	2.45
	SNr		153.16	30.57		134.90	41.02	0.51	0.27	−0.02	1.03

*All SN subdivisions are included*.

*** *Significantly different from controls (p < 0.001)*.

** *Significantly different from controls (p < 0.005)*.

* *Significantly different from controls (p < 0.05)*.

### Comparison Between R2^*^ and QSM

In the eight articles that analyzed both R2^*^ and QSM, Hedge's *g* was significantly higher for QSM than for R2^*^ values (1.16 ± 0.45 vs. 0.55 ± 0.26, *p* = 0.0006).

### Correlation Analyses

Both R2^*^ values and Hedge's *g* did not correlate with either clinical characteristics (age, disease duration, UPDRS, and LEDD) or imaging parameters (voxel size and number of echoes).

For SWI, effect size correlated positively with UPDRS values (*r* = 0.84, *p* = 0.04) and voxel size (*r* = 0.65, *p* = 0.04).

There was a positive correlation between QSM values and age of PD patients (*r* = 0.64, *p* = 0.0001), UPDRS values (*r* = 0.57, p=0.0008) and voxel size (*r* = 0.47, *p* = 0.04).

### Scanner Effects

As expected, R2^*^ values in the SN were lower at 1.5 T (mean 24.45 s^−1^) (9, 17) than at 3 T (mean 38.26 s^−1^), due to the known increase of R2^*^ with magnetic field strength. However, no statistical comparison could be performed due to the low number of data acquired at 1.5 T. In R2^*^, there was a statistical difference between the four vendors (*p* = 0.0006) with Philips providing higher R2^*^ values (mean 50.25 s^−1^) than the other vendors, that is, Siemens (mean 34.68 s^−1^, *p* = 0.0001), Magnex Scientific (mean 34.99 s^−1^, *p* = 0.015), and General Electrics (mean 37.77 s^−1^, *p* = 0.0008) while there was no significant difference between Siemens and General Electrics (*p* = 0.22). There was no longer a statistical difference between the four manufacturers (*p* = 0.93) when R2^*^ values were normalized using control values.

In SWI, there was no between-vendor significant difference in effect size or phase values (*p* = 0.22 and *p* = 0.21, respectively).

In QSM values and Hedge's *g*, there were no between-vendor significant differences (*p* = 0.78 and *p* = 0.07, respectively), and all the studies were similar in ROI definition.

## Discussion

Overall, studies reported in this meta-analysis systematically detected iron overload in the SN by iron-sensitive MRI compatible with PD patients compared to HCs even in the early stages of the disease. This result was in agreement with the abnormal iron metabolism in PD that is associated with the SN cell loss (62).

Studies differed in iron measurement techniques, the studied ROIs, methods of image acquisition and analysis, and patient population. The main measures used to quantify iron load were the R2^*^, the phase and relative phase of the images for SWI, and the susceptibility for QSM. In R2^*^ and QSM, positive values of Hedge's *g* were related to an increase in iron in PD patients as compared to the HCs (6, 7). In SWI, studies used different image analysis techniques while we used Hedge's *g* as an absolute value of the result.

Regarding R2^*^ values, there was a high variability between studies that was due to several factors. First, the age range of PD patients was wide (58 to 72 years). Second, we observed a dependence of the R2^*^ value on the vendor, magnetic field strength, and ROI placement methods. Moreover, previous studies have shown that R2^*^ depends not only on iron content but is also affected by the orientation of the head in the scanner, the surrounding iron distribution (blooming artifacts), the magnetic field strength, and imaging parameters such as the echo time or voxel size (53, 63, 64). Yet all studies demonstrated a significant increase in R2^*^, suggesting that R2^*^ was a robust biomarker of SN changes in PD, especially when using a given protocol on a given scanner. Nevertheless, R2^*^ values in PD became comparable across scanners after normalization by the HC values. Multicenter studies should consider the strong difference in R2^*^ between vendors, especially for Philips, and consider normalizing data.

As for manual or semiautomated segmentation methods, the effect size was higher when the segmentation was performed using anatomical images such as 3D T2-weighted and T2^*^-weighted rather than using R2^*^ or QSM maps, although the difference was not statistically significant. This difference may be related to the better resolution of the anatomical images. However, the constant improvements in resolution and contrast of parametric mapping, especially at ultra-high-field strength, especially in QSM, may modify this observation in future studies. There were no significant correlations for R2^*^ values or for R2^*^ effect size with clinical parameters. The lack of correlations may be due to the large variability of R2^*^ results as well as to a high number of possible confounding factors. Finally, while most articles reported UPDRS values, a large proportion of them did not specify if the values were obtained using on or off medication. In PD, degeneration of dopaminergic neurons predominates in the lateral part of the SNc, particularly in nigrosome 1 (65); therefore, greater differences were expected in this region. The highest effect size was observed in this lateral part of the SN consistent with this hypothesis. Some authors have analyzed separately the SN contra and ipsilateral to the most affected side in the hypothesis that the contralateral SN would show greater nigral damage (12, 31, 32). However, we found no difference for both R2^*^ values and effect sizes. This suggests that at PD onset, both SNc and SNr are already affected with increased iron overload.

SWI measurements were the most variable across studies. SWI is purely a qualitative method that some studies have however used to carry out local measurements (66). However, this is intrinsically incorrect, since these phase measurements do not reflect local modifications because the phase shift of the signal inside a voxel comes not only from sources of susceptibility inside this voxel but also from neighboring sources outside this voxel (67). This can be a reason for the high variability across studies (I^2^ = 89%), even if the pooled effect size was rather high. Additional reasons for the variability of SWI values in these studies can be the presence of blooming artifacts on phase and SWI images (similar to R2^*^) and the dependence of SWI on tissue geometry and orientation dependence relative to the direction of the main magnetic field. Limitations of SWI induced by the nonlocal, geometry-dependent, and orientation-dependent nature of the signal phase are overcome by QSM, which directly estimates the tissue susceptibility distribution based on the local perturbation of the magnetic field (64). Still, there was a positive correlation between the UPDRS scores and the effect size of phase measurements, suggesting a correlation between iron load and the severity of motor symptoms.

The effect size observed for QSM was higher than the one for R2^*^ although the difference was not significant. Moreover, in studies that compared R2^*^ and QSM values in the same patients, the effect size was significantly higher for QSM than for R2^*^, suggesting that QSM might be a more robust marker than R2^*^. In addition, there were no significant differences in QSM values between the vendors. The QSM values also correlated significantly with age and UPDRS. The variability of susceptibility measurements was lower than that for SWI; however, this variability was still high, suggesting that more work needs to be done to standardize QSM image processing pipelines between centers. There were no significant differences in QSM values between the different subdivisions of the SN or between the SN contra or ipsilateral to the most affected side. Overall, while we would expect more significant changes in the SNc than the SNr based on pathological studies, this difference was not found in a high proportion of studies. This lack of difference could be due to the segmentation techniques, since the SNc was not clearly visible on the R2 ^*^ or QSM images due to the relatively weak presence of iron in this region and its segmentation most often using a probabilistic method. A neuromelanin-based segmentation method could help better delineate the SNc (56).

Finally, the results of this meta-analysis suggest that QSM has some advantages over R2^*^ because it provides a quantification of magnetic susceptibility, which might better reflect the underlying tissue iron content compared to R2^*^. Moreover, both QSM and R2^*^ are preferable to SWI because they provide quantitative values unlike SWI.

In terms of implementation, however, all methods pose some issues. Firstly, QSM and SWI require acquiring images of the MRI signal phase, whereas R2^*^ only requires acquiring images of the MRI signal magnitude. Although protocols for SWI are now available for most vendors, for QSM, the correct acquisition of the phase might pose a technical issue in a clinical setting, as not all vendors correctly combine the phase from multiple elements of a phased-array coil, and dedicated software is required in these cases (68). Secondly, SWI only requires a single-echo acquisition, whereas multi-echo acquisitions are required for R2^*^ and are in general preferable for QSM too (69–71). This might pose a time issue in clinical practice. Therefore, when deciding which methods to use, one should consider the need for quantitative vs. qualitative imaging, the ease of implementation on clinical systems, and time constraints.

Our study has several limitations. First, despite the careful search across several databases, some studies could have been missed (72). Second, common with all literature searches and meta-analysis publication practices, usually publishing positive rather than negative studies might have biased the results. Consequently, the mean effect size could be somehow overestimated as unpublished negative data were probably underrepresented. Third, there was a large heterogeneity of R2^*^ and SWI values, indicating a large variability of these measurements, that depended on blooming artifacts and the fact that, in contrast with QSM, neither R2^*^ nor SWI directly quantifies the changes in magnetic susceptibility due to iron deposition in the SN. Moreover, the values varied with the scanner vendor and other technical parameters.

In summary, we have observed a consistent increase in MRI measures of iron content in PD across the literature using R2^*^, SWI, or QSM techniques, confirming that these measurements provide reliable markers of iron content in PD. Several of these measurements correlated with the severity of motor symptoms. Lastly, QSM appeared to be a more robust biomarker than R2^*^. However, image processing pipelines for QSM are not yet fully standardized, although efforts in this direction are being made (68, 73, 74). Therefore, QSM is a promising biomarker of disease-related iron accumulation in PD, but further work is needed to establish it as a robust biomarker in multicenter clinical studies and its usefulness as a longitudinal marker.

## Data Availability Statement

All datasets generated for this study are included in the article/Supplementary Material.

## Author Contributions

NP, CS-M, and SL contributed conception and design of the study. NP, RG, and CS-M organized the database. LY-C performed the statistical analysis. NP wrote the first draft of the manuscript. NP, CS-M, EB, MS, and SL wrote sections of the manuscript. All authors contributed to manuscript revision, read, and approved the submitted version.

## Conflict of Interest

The authors declare that the research was conducted in the absence of any commercial or financial relationships that could be construed as a potential conflict of interest.

## Supplementary Material

Supplementary Figure 1 Prisma 2009 flow diagram showing an overview of study selection.

Supplementary Figure 2 Funnel plot of R2^*^ values.

Supplementary Figure 3 Funnel plot of SWI values.

Supplementary Figure 4 Funnel plot of QSM values.

Supplementary Figure 5 Forest plot of significant R2^*^ values of the 2*7* articles included in the meta-analysis. Forest plot of the computed diseases effect sizes (Hedge's g, x-axis) of studies included into the meta-analysis on R2^*^ measures of the substantia nigra when comparing PD patients and controls. Pooled SMD (95%) *(0.84, [0. 60, 1.08])* is denoted by a blue diamond.

Supplementary Figure 6 Forest plot of significant *QSM* values of the *16* articles included in the meta-analysis. Forest plot of the computed diseases effect sizes (Hedge's g, x-axis) of studies included into the meta-analysis on *QSM* measures of the substantia nigra when comparing PD patients and controls. Pooled SMD (95%) (*1.13*, [*0.86, 1.39*]) is denoted by a blue diamond.

Supplementary Table 1 PRISMA checklist.

Supplementary Table 2 Quadas questionnaire.
