# Supplementary figures and images for: Iron Imaging as a Diagnostic Tool for Parkinson's Disease: A Systematic Review and Meta-Analysis
Source: Front Neurol. 2020 May 28;11:366. doi: 10.3389/fneur.2020.00366 (PMC7270360; doi:10.3389/fneur.2020.00366)

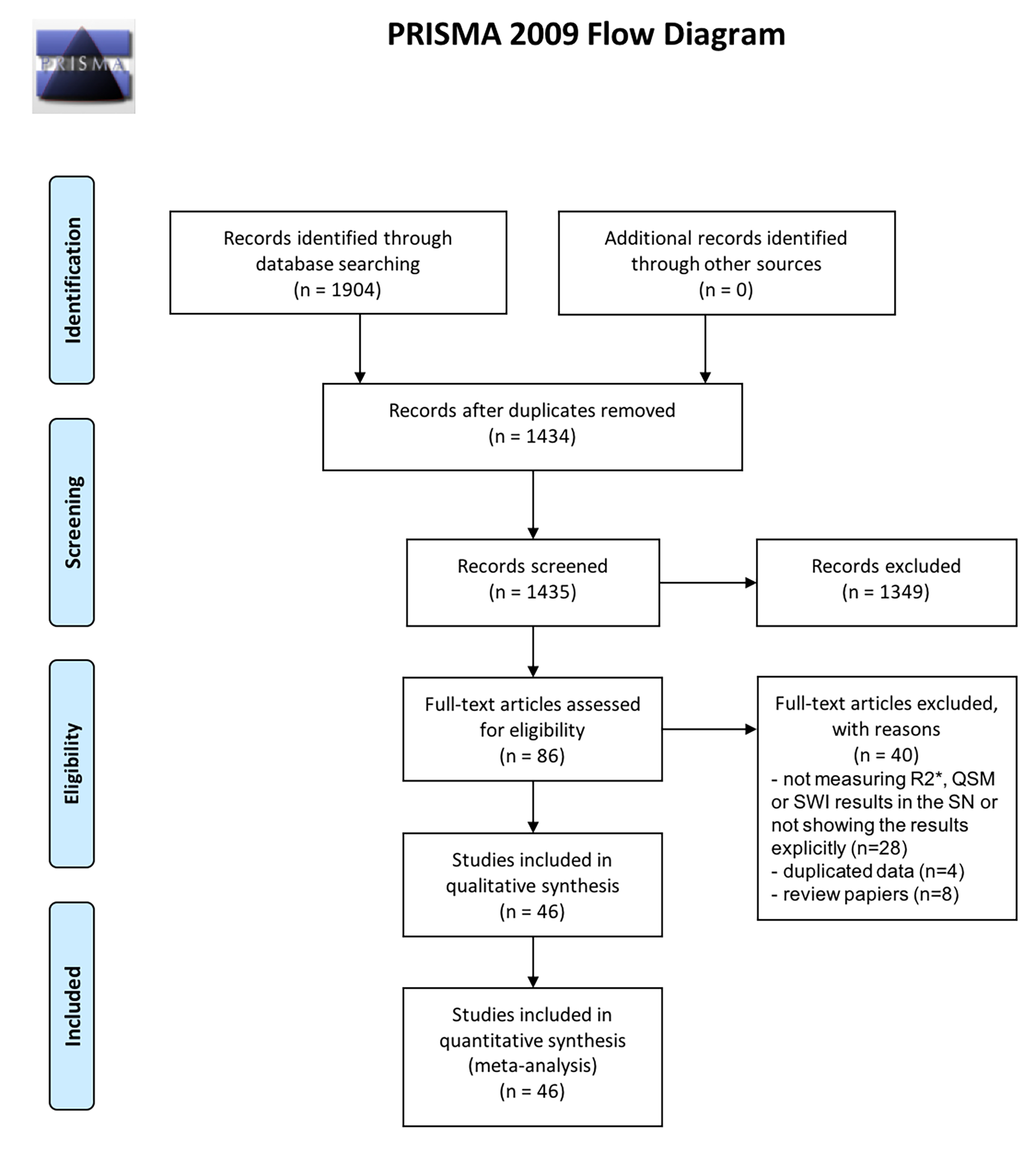

Supplement: Supplementary Figure 1 — Prisma 2009 flow diagram showing an overview of study selection. [file Image_1.TIF]

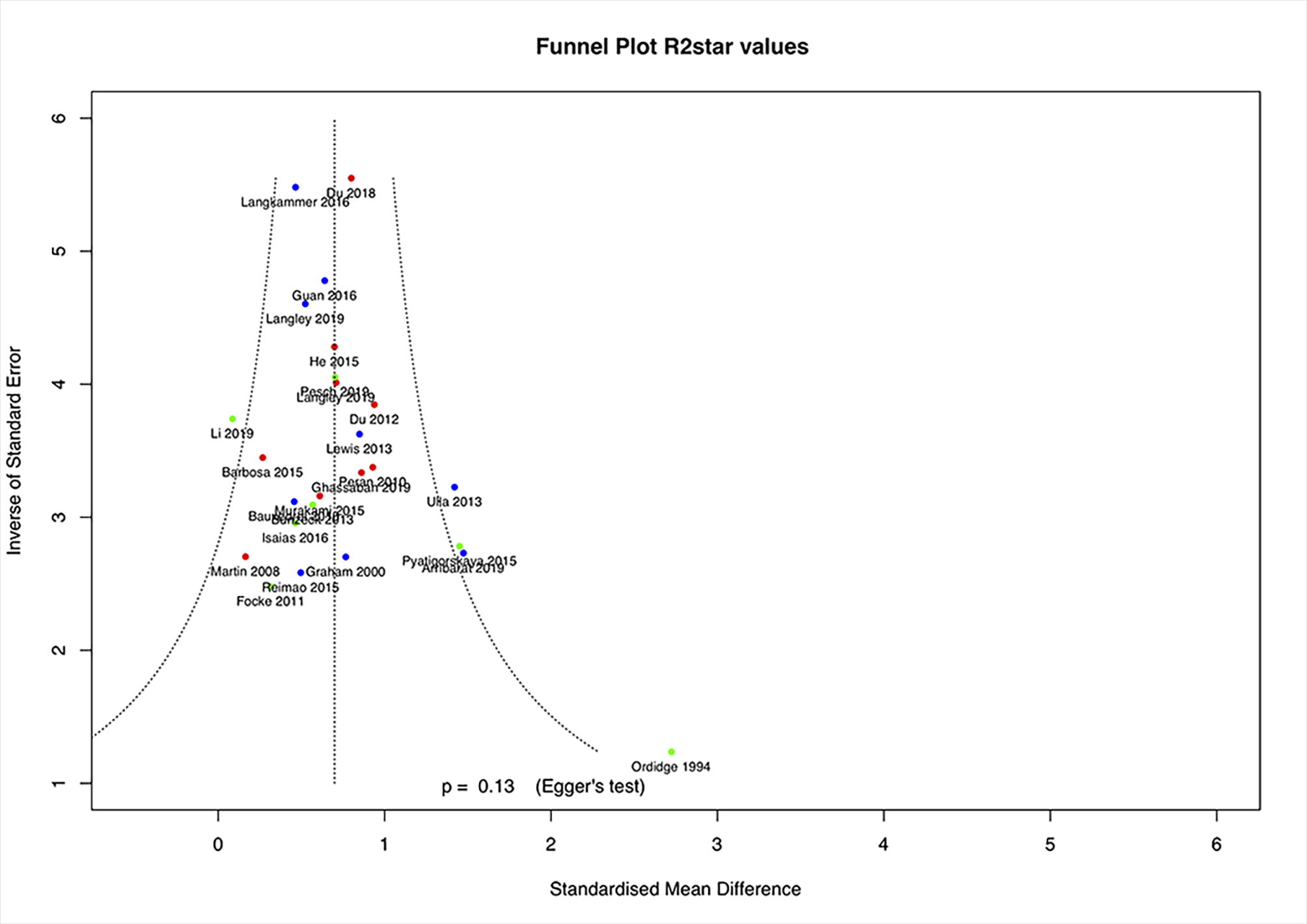

Supplement: Supplementary Figure 2 — Funnel plot of R2* values. [file Image_2.TIFF]

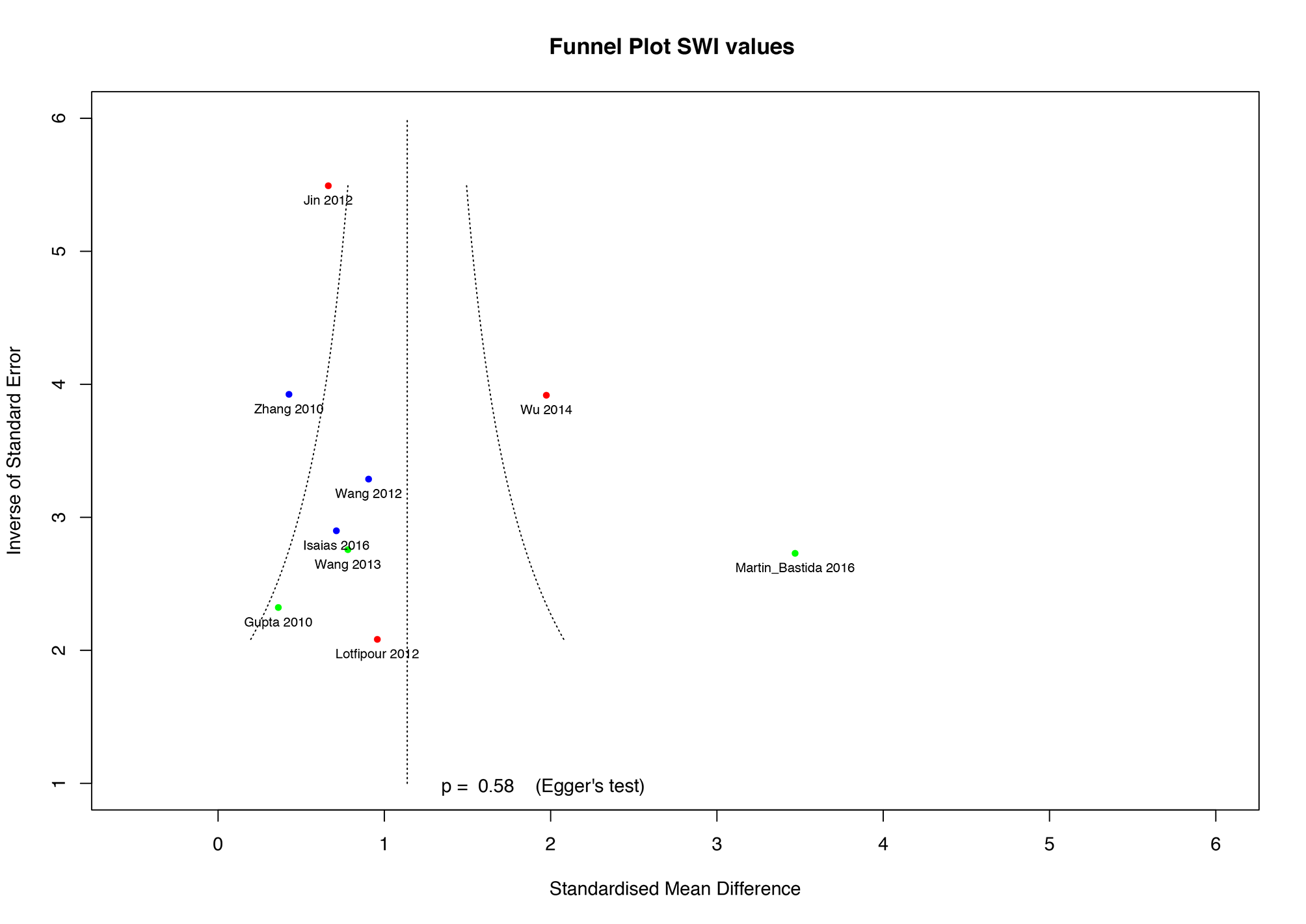

Supplement: Supplementary Figure 3 — Funnel plot of SWI values. [file Image_3.TIF]

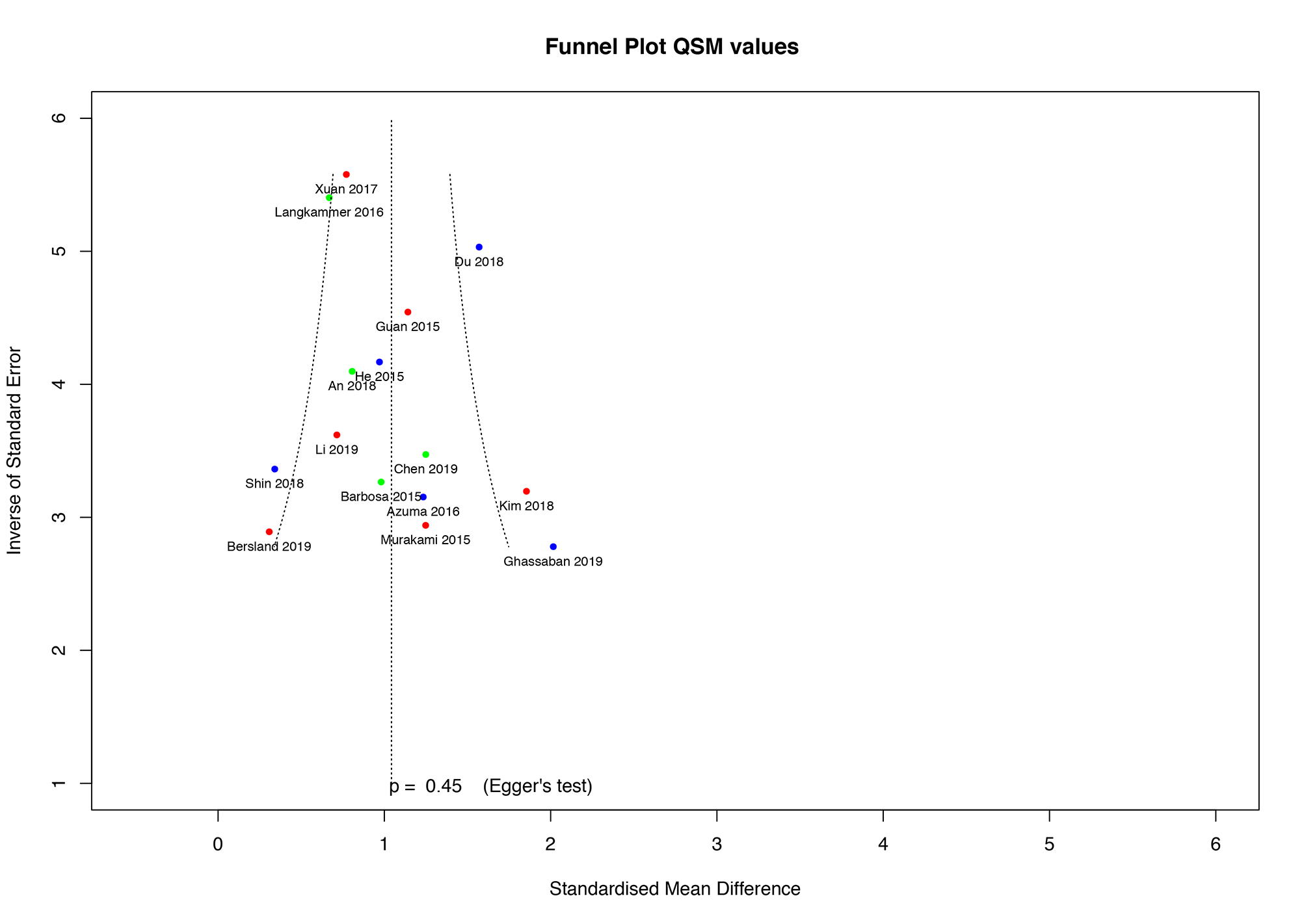

Supplement: Supplementary Figure 4 — Funnel plot of QSM values. [file Image_4.TIF]

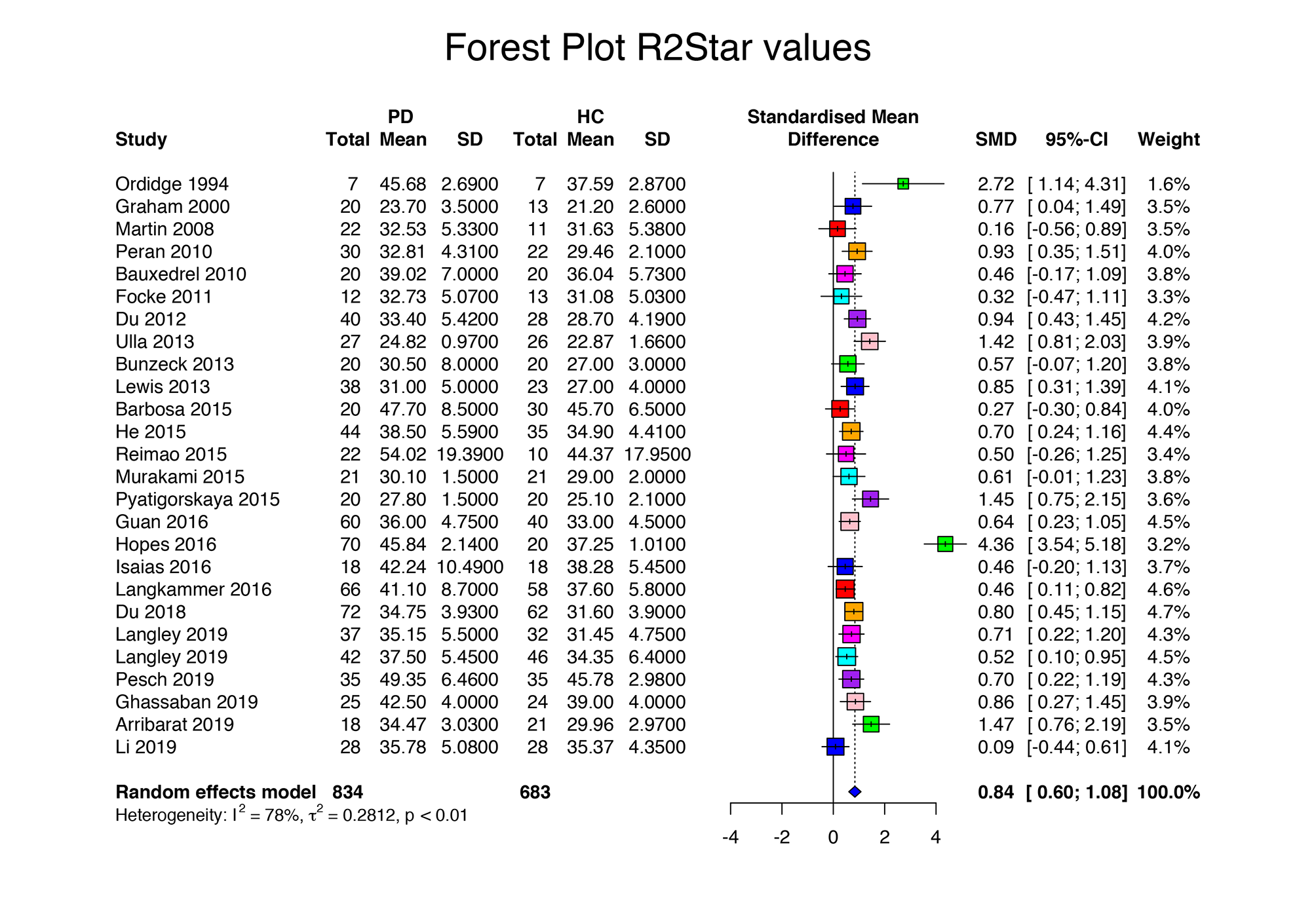

Supplement: Supplementary Figure 5 — Forest plot of significant R2* values of the 27 articles included in the meta-analysis. Forest plot of the computed diseases effect sizes (Hedge's g, x-axis) of studies included into the meta-analysis on R2* measures of the substantia nigra when comparing PD patients and controls. Pooled SMD (95%) (0.84, [0. 60, 1.08]) is denoted by a blue diamond. [file Image_5.TIF]

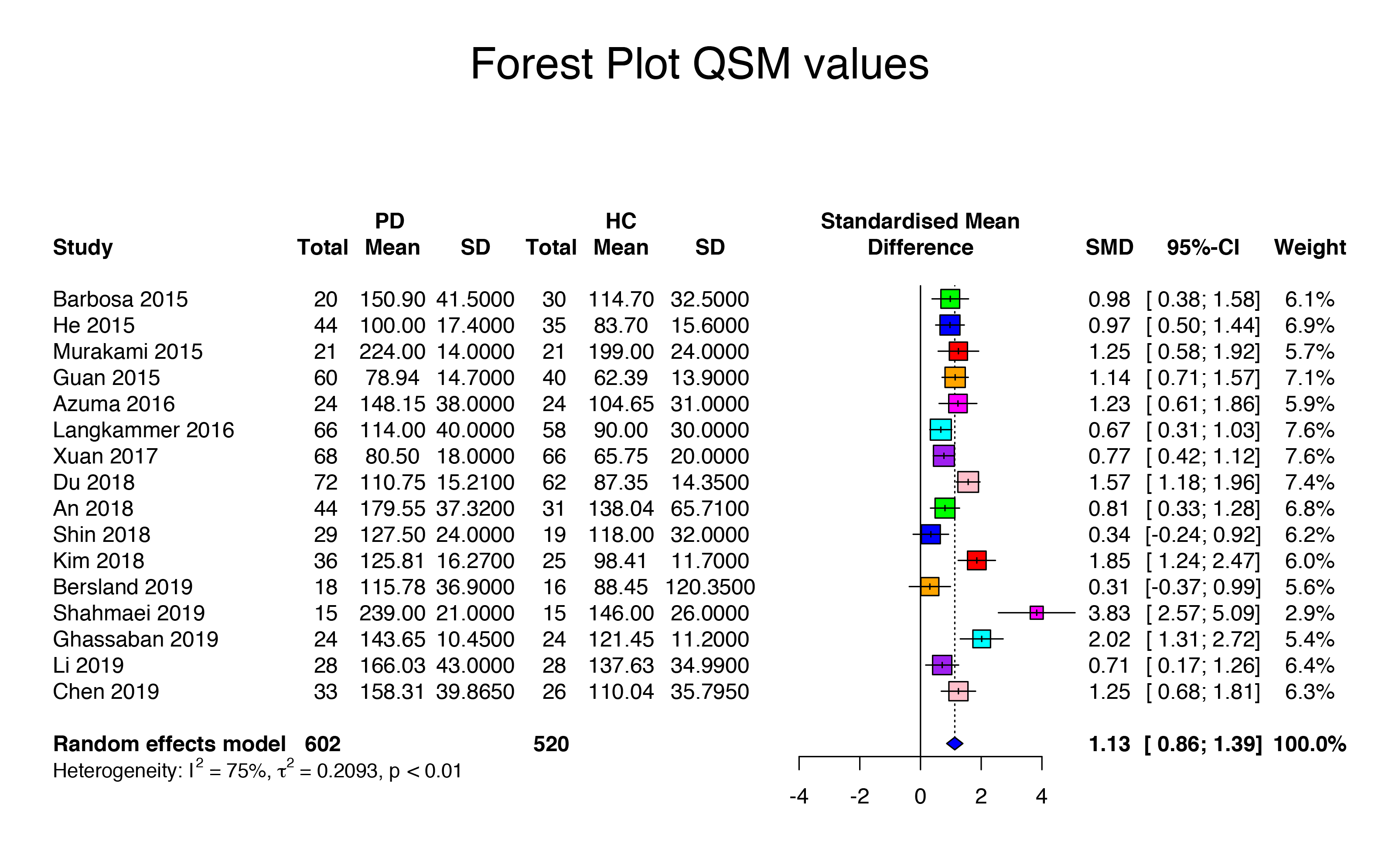

Supplement: Supplementary Figure 6 — Forest plot of significant QSM values of the 16 articles included in the meta-analysis. Forest plot of the computed diseases effect sizes (Hedge's g, x-axis) of studies included into the meta-analysis on QSM measures of the substantia nigra when comparing PD patients and controls. Pooled SMD (95%) (1.13, [0.86, 1.39]) is denoted by a blue diamond. [file Image_6.TIF]
